# Supplementary material for: Protein Microarrays for High Throughput Hydrogen/Deuterium Exchange Monitored by FTIR Imaging
Source: Int J Mol Sci. 2024 Sep 16;25(18):9989. doi: 10.3390/ijms25189989 (PMC11432650; doi:10.3390/ijms25189989)
Supplement: Supplementary file 1 [file ijms-25-09989-s001.zip › ijms-3181509-supplementary.pdf]

Supplementary materials for

High throughput real time measurement of HD exchange in proteins by FTIR spectroscopy of protein microarrays.

Joëlle De Meutter and Erik Goormaghtigh

Center for Structural Biology and Bioinformatics, Laboratory for the Structure and Function of Biological Membranes, Campus Plaine CP206/02; Université Libre de Bruxelles, B1050 Brussels, Belgium

Contents

Microarray data pre-processing (Figures S1-S8) 2
Kinetic analysis (Figures S9-S11) 7
Figure S12a : inverse Laplace transform of the HDX curves (proteins 1-24) 11
Figure S12b : inverse Laplace transform of the HDX curves (proteins 25-48) 12
Figure S12c : inverse Laplace transform of the HDX curves (proteins 49-72) 13
Figure S12d : inverse Laplace transform of the HDX curves (proteins 73-85) 14
Figure S13: curve fitting of the 85 proteins by 3 or 4 time constants 15
Figure S14A: curve fitting results for 3 time constants 16
Figure S14B: curve fitting results for 3 time constants, forced to the mean 17
Figure S14C: curve fitting results for 4 time constants 18
Figure S15: correlation between exchange rates and secondary structure content 19
Table S1: list of the proteins and their characteristics 20
Table S2: time constants obtained by inverse Laplace transform for all proteins 22
Table S3: time constants obtained by curve fitting for all proteins 24
References 27

Microarray data pre-processing (Figures S1-S8)

HDX measurements were obtained as a series of about 30 FTIR images recorded as a function of the time of exposure to the ^2H_2O vapor. The first image was recorded just before connecting the ^2H_2O-saturated N_2 flow to the microarray cell. Nitrogen was bubbling in 3 vials (assembled in series) at a rate of ca 80 ml N_2 gas/min. 64 scans were recorded for each image, which takes about 2 minutes. About one additional minute was needed to transfer the data to the computer and get the spectrometer ready for the next measurement. The recording time reported here is the average between the beginning of the scanning and the end. During the first hour, images were recorded continuously, i.e. about one image every 3 minutes. After the first hour, the time interval between 2 measurements was larger. An example of time series is (time in min):

0.0	1.2	4.3	7.5	10.4	13.3	16.9	20.1	23.5	26.8	29.7	33.3
36.7	39.6	42.7	46.1	49.4	55.8	59.1	61.9	68.1	78.2	111.1	173.1
235.0	290.0	350.0	409.0	414.0	1411.0	1446.0					

The actual time points were recorded independently for each experiment.

The processing of the data, fully automated in the Kinetics program, is described below step-by-step below.

1. Load the image

Figure S1 presents the absorbance at 1654 cm^{-1} of a protein microarray. The data cube contains 128x128 spectra recorded between 3900 and 900 cm^{-1}, encoded every 2 cm^{-1}.

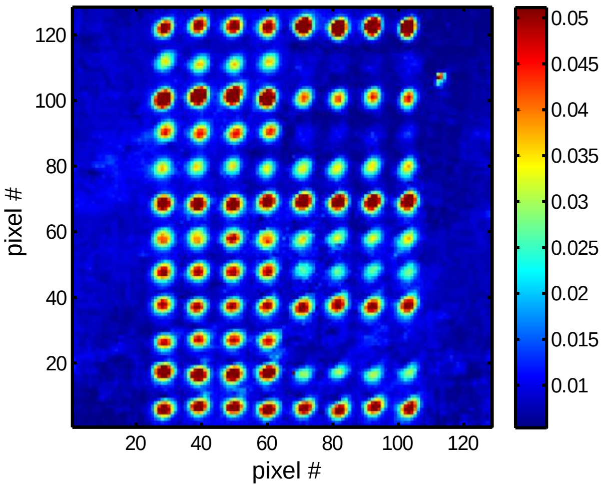

Figure S1: absorbance at 1654 cm^{-1} of a protein microarray containing 96 protein spots. The color bar reports the absorbance color scale.

2. Define a SNR threshold

The signal-to-noise ratio (SNR) allows the identification of the spectra characteristic of the proteins, originating from the spots, versus the spectra originating from empty regions, between the spots. For each of the 16,384 spectra, two values are computed

1. The standard deviation of spectral region between 2,000 and 1,900 cm^{-1}. In this spectral region, no contribution from the protein is expected. This standard deviation is therefore a measure of the noise.
2. The “Signal” is evaluated as the highest absorbance found above a baseline drawn between 1760 and 1480 cm^{-1} as illustrated in Figure S2.

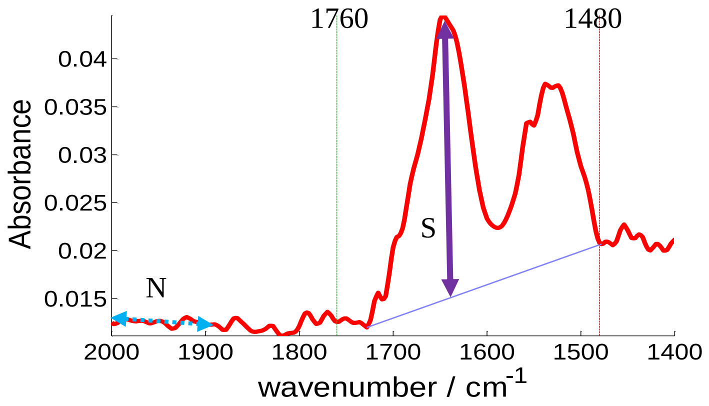

Figure S2: illustration of the evaluation of the signal (S) and of the noise (N). A baseline is drawn between the local minima found around 1760 and 1480 cm^{-1}.

It can be observed that the spectrum presented in Figure S2 is still of poor quality, with obvious contributions from water vapor overlapping the protein spectrum. It can also be observed that the exact level of the baseline can be affected by the water vapor contribution.

A threshold of SNR=35 allows the identification of the protein spots. In Figure S3, the background, i.e. pixels with spectra where SNR<35, has been colored in black.

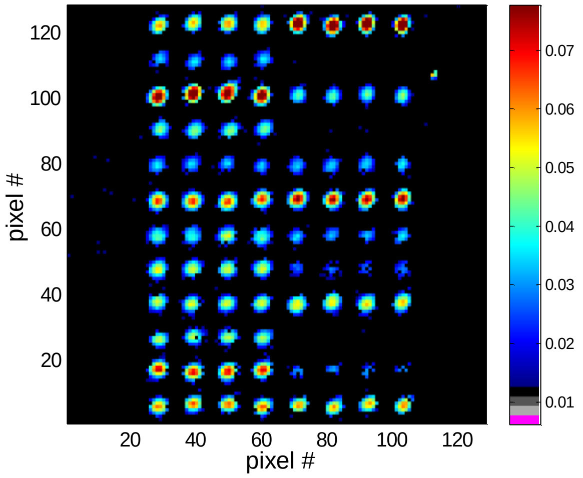

Figure S3: absorbance at 1654 cm^{-1} of a protein microarray containing 96 protein (or control) spots. Pixels where SNR<35 have been colored in black. The color bar reports the absorbance color scale.

3. Subtract a general background

A major advantage of microarrays is that a very good background recorded in the same experimental conditions and exactly at the same time as the protein spectra is available between the spots. A first general background is defined therefore as the mean spectrum of a series of spectra found outside the microarray range.

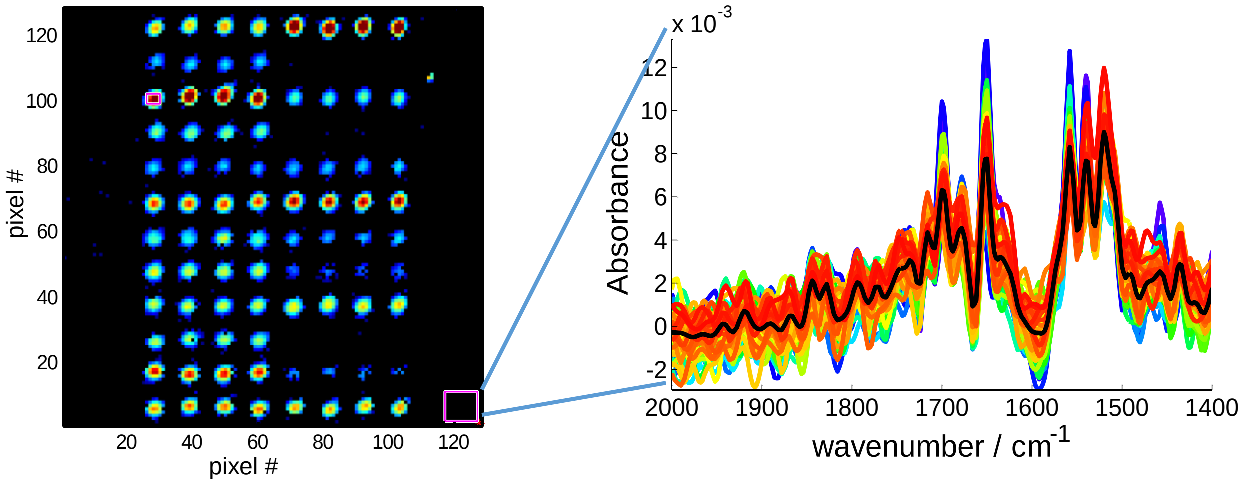

Figure S4: Left: absorbance at 1654 cm^{-1} of a protein microarray containing 96 protein spots. Pixels where SNR<35 have been colored in black. A magenta rectangle has been drawn at the right bottom of the microarray. Right: series of 110 spectra collected within the magenta rectangle drawn on the left part of the figure. The mean of these 110 spectra appears in black.

In Figure S4, a rectangle has been drawn at the right bottom of the microarray, defining a series of 110 spectra recorded outside of the microarray area. It can be trusted they represent all contributions, including noise, that do not originate from the proteins. Water vapor is predominant. The mean of the 110 selected spectra is then calculated. This mean spectrum can now be subtracted from all the spectra of the image, therefore correcting for these contributions that are unrelated to the sample to be analyzed. This first correction is necessary for a precise definition of the SNR. The water vapor contributions that overlap the amide I band could indeed be confused for protein signal. This is particularly critical for diluted proteins with low absorbance peaks when searching for the signal as depicted in Figure S2.

4. Apply grids to identify the protein spots and local background subtraction

In the microarray illustrated above, 4 replicates of the same proteins have been spotted horizontally. There is therefore 2 different proteins in quadruplicates on each line of the microarray. Two different grids (Figure S5) are therefore required for automation of the analysis. They are shown in Figure S5. The first one (white) has 12 rows for 12 different proteins (or controls) and 4 columns for the quadruplicates. Similarly, the second grid (magenta) has 12 rows and 4 columns.

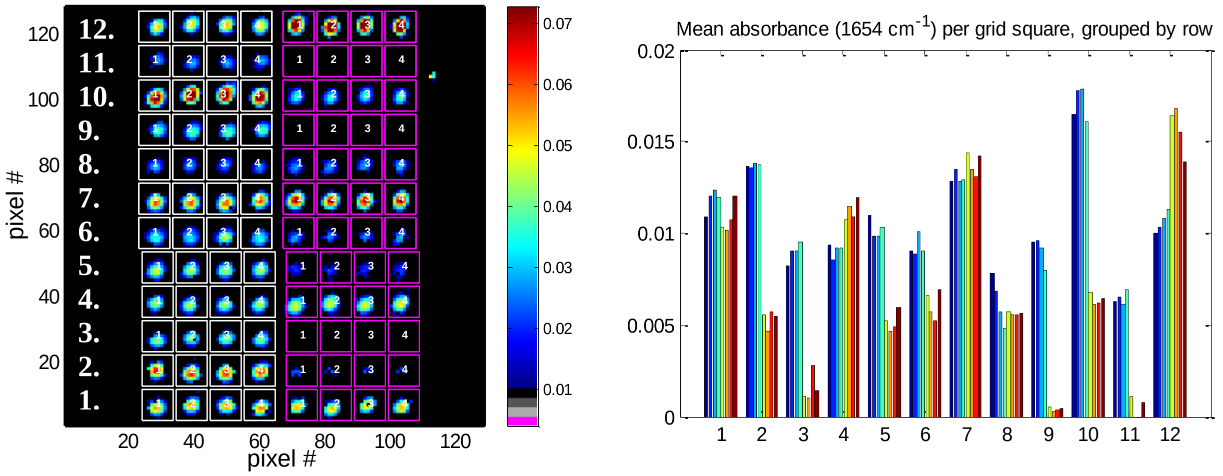

Figure S5: Left. Absorbance at 1654 cm^{-1} of a protein microarray containing 96 protein spots. Pixels with SNR<35 have been colored in black. The absorbance scale is provided by the color bar. Numbers in the left margin identify the 12 proteins of grid #1. Right. Mean absorbance of each protein spot in the 12 rows, starting from the bottom. Blue bars are for the 4 spots of grid #1, red bars for the spots of grid #2.

Now that the general background has been subtracted, a further improvement in the quality of the spectra can be obtained by subtracting the local background of each spot, i.e. for each spot, the mean of the spectra that appear as black pixels (Figure S5) in the square containing the spot.

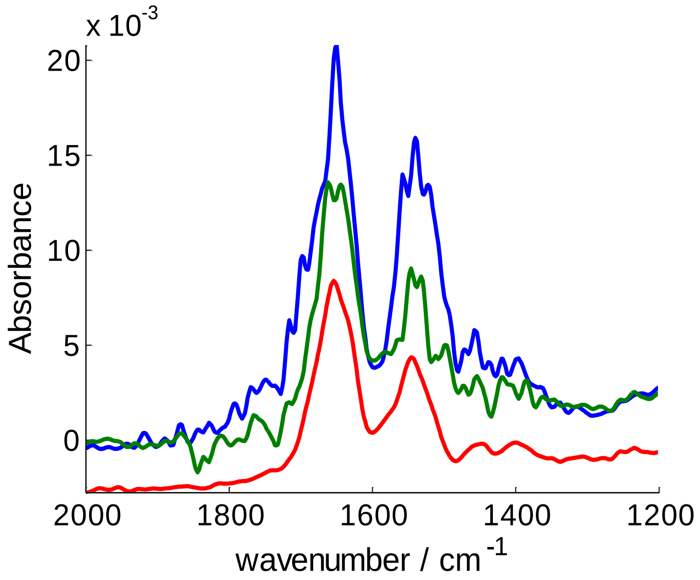

Figure S6: Mean spectra of 42 spectra collected in the weak spot located around pixel at x=28, y=112 visible in Figure S5 (second row, last column) before any processing (blue curve), after subtraction of the general background as indicated in Figure S4 (green curve) and after further subtraction of the background present in this particular square (red curve)

It is clear from Figure S6 that, for a low intensity protein signal, the two levels of subtraction are necessary.

5. Microarray spectrum collection

Once the cube of data has been processed as indicated above, for each square of each grid, the mean spectrum of the spectra passing the SNR threshold are computed and saved. Figure S7 illustrates the result for grid #1 shown in Figure S5.

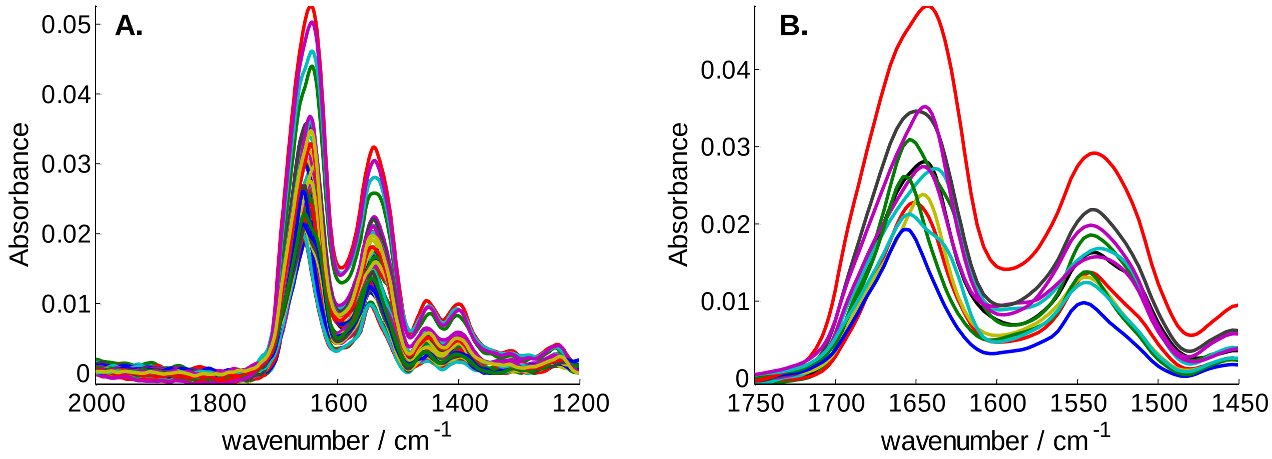

Figure S7: A. 48 mean spectra of the 48 squares of grid #1 (Figure S5) and B. zoom between 1750 and 1450 cm^{-1} on row-wise averaged spectra of grid #1 presented in Figure S5, leaving 12 averaged spectra.

While the whole process may appear tedious, it is in fact completely automated. A macro implemented in the Kinetic software reads the FTIR image files one by one, does all the processing steps described above and save the spectra as illustrated in Figure S7. As there are about 30 time points in a HDX kinetic

experiment and 96 spots on each grid, 2880 spectra are generated. In addition, the Kinetics software also generates mean spectra for each row of each grid, adding 720 mean spectra to the previous series. Handling kinetic data is therefore a challenge. It is solved by including information in the file names according to the following sequence: 1) the grid number when there are several grids as in Figure S5, 2) the square number and finally 3) the image number, starting at 1 for the first time point and incremented by 1 for each increasing time of exposure to ^2H_2O vapor. Sorting the names alphanumerically results therefore in the same sequence, 1) all spectra from the same grid, 2) within this grid, all spectra of a same protein and 3), for each protein, the different images sorted according to the exposure time to ^2H_2O. As an example, spectra collected for the 12 proteins present in grid#1 of Figure S5, at all time points, are shown in Figure S8.

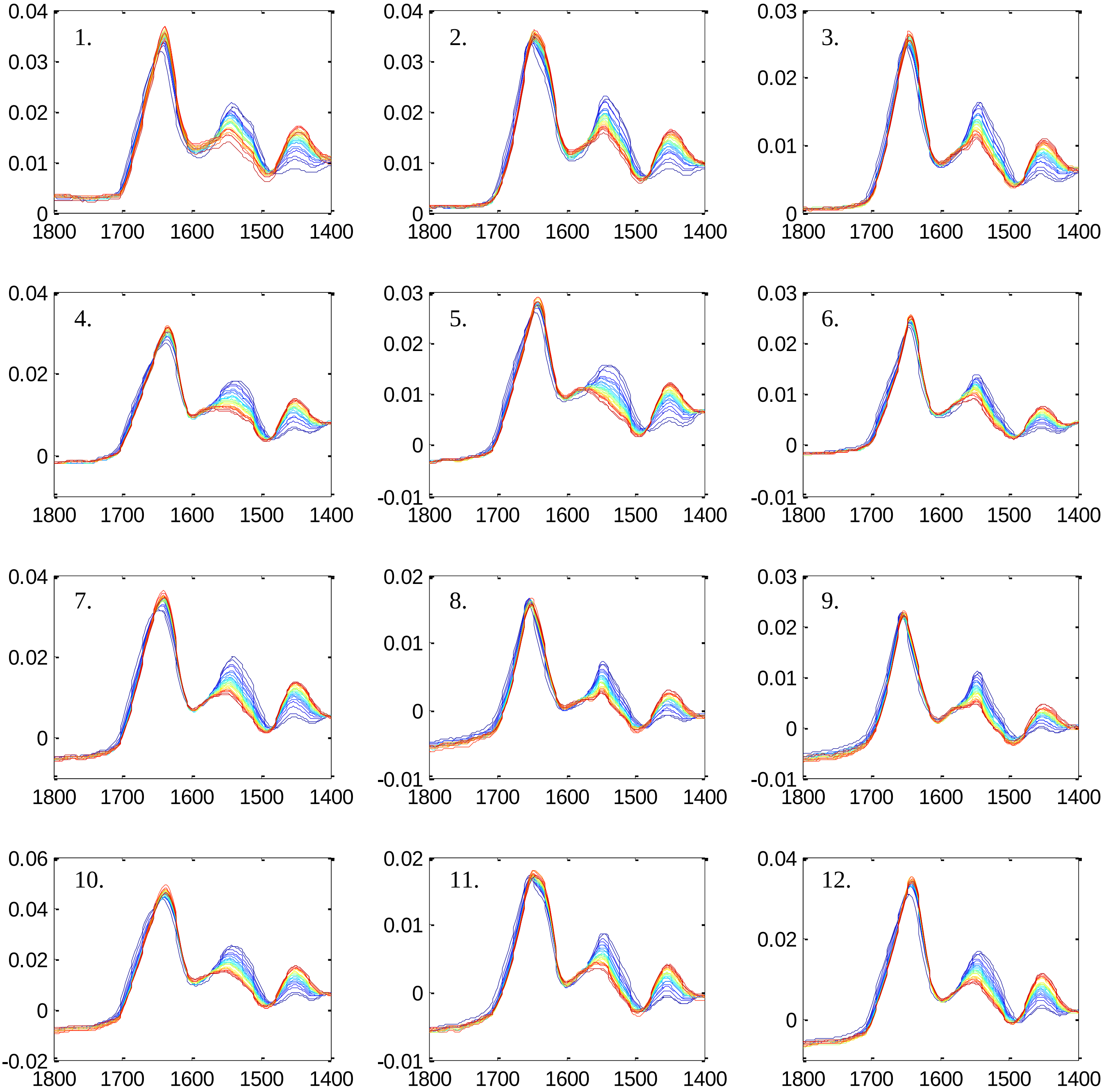

Figure S8: 12 mean spectra (row-wise) of the 48 squares of grid #1 (Figure S7) recorded as a function of the time of exposure to ^2H_2O. Time scale is indicated by the color, from blue at time=0 to red after overnight deuteration. Data in this figure are sorted from left to right, from top to bottom. Numbering corresponds to the numbering indicated in the left margin of Figure S5A.

Kinetic analysis (Figures S9-S11)

Integration of amide I – amide II areas

Once the spectra have been collected and sorted alphanumerically, another macro implemented in Kinetics cycles automatically on all the spectra of each protein and computes the area of amide I, amide II and amide II' for all the time points. The experimental time points are requested by the macro at the beginning. For integration of amide I, amide II and amide II', a linear baseline is drawn between spectral points delimiting these bands. For each of these limits, the program searches for a local minimum within a margin that can be specified, here 10 cm^{-1}. The selection of such baseline for integration results in a near zero absorbance of amide II after full deuteration that occurs in fully disordered proteins or after denaturation of protein secondary structure. It is the expected behavior of amide II that is supposed to disappear completely when exchange of amide N-H is complete. Considering integration down to the zero level does not result in satisfactory results, largely because of side chain contributions present in this part of the spectrum. The baseline shown on Figure S9 in fact roughly accounts for the side chain contributions, in line with published analyses of side chain contributions in the amide I –amide II region of the spectrum.(Chirgadze et al. 1975; Goormaghtigh et al. 1994, 1996; Raussens et al. 1996, 2004; Rahmelow et al. 1998; Hollecker et al. 2002; Barth 2007; Goormaghtigh 2009; De Meutter and Goormaghtigh 2021) An example of integration is reported in Figure S9.

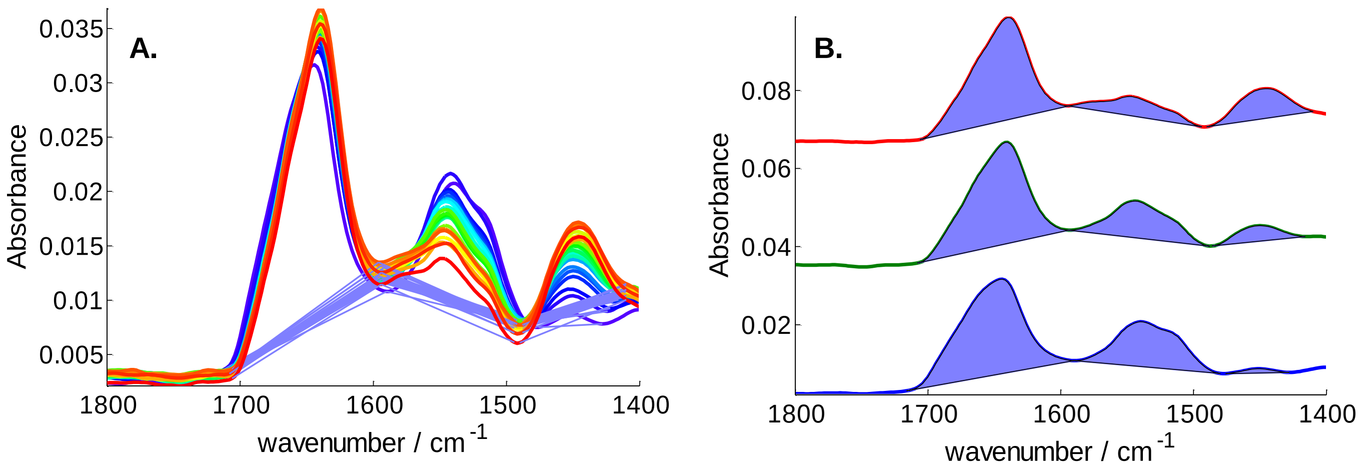

Figure S9: A. series of spectra recorded as a function of the deuteration time. In this example, spectra of protein #1 identified in Figure S5 and S8 is represented. Time scale is indicated by the color, from blue at time=0 to red at the end of the process. Baseline used for integration are shown, B. example of integration for spectra recorded at time 0 (bottom), 15 min (middle) and overnight (top). Spectra have been offset for the sake of the clarity.

Integration generates data in the following format:

M_grid 2_04_RawMeanSp.res			
Time (min)	AmI area 1720-1595 ±10 - ±10	AmII area 1590-1485 ±10 - ±10	AmII' area 1485-1420 ±10 - ±10
0	1.3591	0.63415	0.02585
2.18	1.3476	0.6076	0.05583
4.52	1.3136	0.54375	0.08215
8.08	1.3258	0.51276	0.10759
11.39	1.3344	0.49226	0.13312
15.27	1.3175	0.48273	0.14681
18.39	1.3217	0.45273	0.16375
22.22	1.3525	0.45968	0.18203
25.34	1.3598	0.44788	0.19475
28.4	1.3281	0.42727	0.20072
31.45	1.3074	0.41031	0.20310
35.09	1.3227	0.41496	0.21871
etc.			

Fragment of a table reporting the area of amide I, amide II and amide II'. The areas have been calculated between the limits reported (in cm^{-1}) in the second row of the table. The margins of ±10 cm^{-1} reported under each limit indicate that the actual limit used to draw the baseline was the minimum of the curve found around the limit ±10 cm^{-1}.

After integration, one such table is generated by the macro for each protein spot and for the mean of each row in a given grid.

2. Kinetic analysis by curve fitting

As protein "concentration" is not precisely defined, analysis of the integration data requires a normalization. This is obtained by dividing the area of amide II by the area of amide I which is supposed to remain constant in the course of the HDX kinetics. Zero time is set as 100% ^{1}H form and zero amide II area is set as 0% ^{1}H form for the amide N-H.

The data are then fitted by a series of 3 exponential decays. The approximate conditions for starting the curve fitting are 3 time constants representing the fast exchanging amides (t_1=1 min), the medium exchange rate (t_2=20 min) and the slowly exchanging amide (t_3=1000 min) and 3 proportions, all set here at 33%. These decay time constants and the proportion of the 3 populations are adjusted by the least-square curve fitting. In the present work, those values have been adjusted as indicated in the paper.

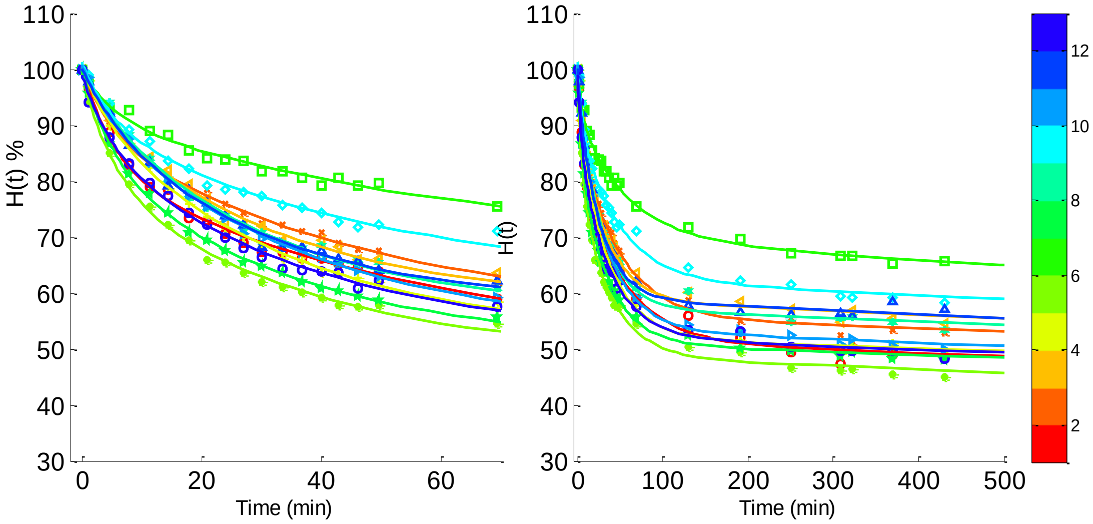

Figure S10: Exchange curves for the series of 12 proteins reported in Figure S8, corresponding to the 12 proteins present in grid #1 of Figure S5. The protein number (row number in Figure S5) are identified by the color reported on the color bar on the right hand side of the figure. Fitting was performed with 3 exponentials. The left panel presents the data between 0 and 70 min, the right panel presents the data between 0 and 500 min.

Results of the fitting are provided as equations, for instance for protein 1 of Figure S5:

$$2.71\ e^{-t/4.99}+12.37\ e^{-t/25.37}+85.67\ e^{-t/10000}, \text{ standard deviation of the fit } 1.74 \quad (1)$$

3. Kinetic analysis by inverse Laplace Transform

An alternative to curve fitting is to compute the inverse Laplace transform of the exchange curve. The inverse Laplace transform computed according to regularized approach developed by Provencher. (Provencher and Dovi 1979; Provencher 1982) The most parsimonious result is obtained, often yielding 3 or 4 main categories of amide protons. Such a distribution is presented in Figure S11.

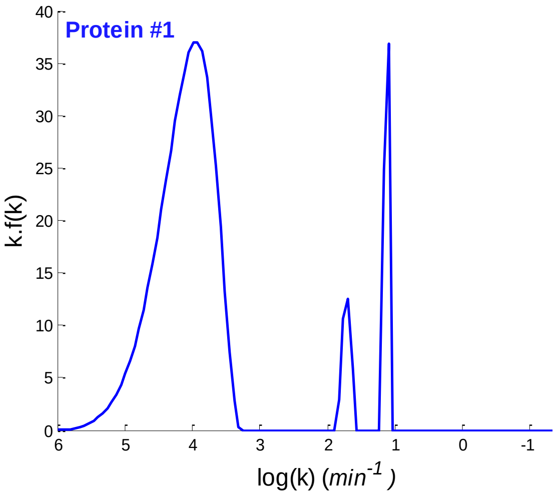

Figure S11: Inverse Laplace transform of the HDX curve obtained for protein #1 of Figure S5

Reproducibility of exchange curves

In Figure S5, grid #1, 12 proteins have been spotted in quadruplicates, making 48 samples available. Fitting of the 48 samples is presented in Figure S12A. Comparing the reproducibility is not possible if both the times constants and the proportion of amide in each class are different for each sample. For the sake of the comparison, a first fitting is run with both time constants and proportions let free to adjust.

One way to circumvent this issue is to average the time constant found for each replicate of a protein and run a second fit constraining these time constants to their mean values, i.e. only the proportion of amide groups in each class is allowed to vary.

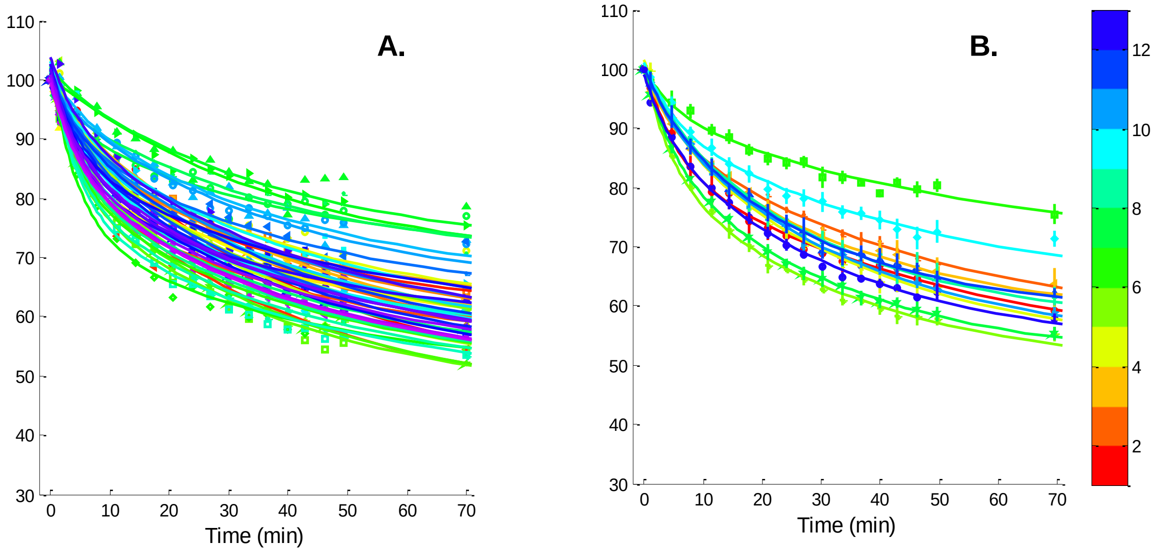

Figure S12: A. HDX curves for quadruplicates measurements obtained on the 12 proteins of grid#1 displayed in Figure S5. B. Mean of the quadruplicate data for the 12 proteins. The error bars represent the standard deviation. Protein number can be identified by the color scale bar on the right hand side of the figure.

In Figure 12A., the quadruplicates of HDX curves for 12 proteins is presented. Figure S12B reports the mean of the HDX data for each protein along with a standard deviation and the fit of the mean data obtained after constraining the time constants to the mean of the value found during the first fitting.

Figure S12a : inverse Laplace transform of the HDX curves (proteins 1-24)

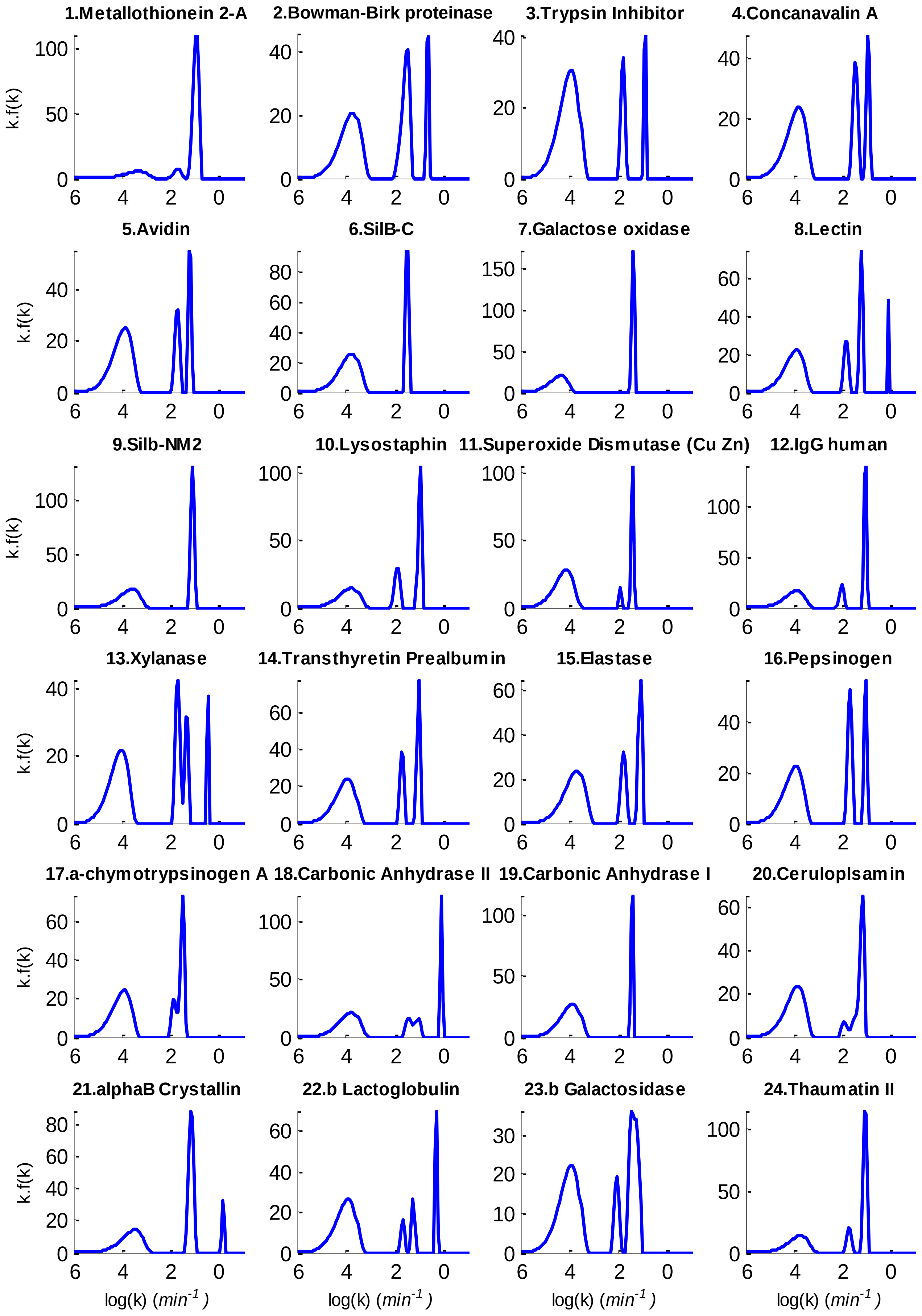

Figure S12b : inverse Laplace transform of the HDX curves (proteins 25-48)

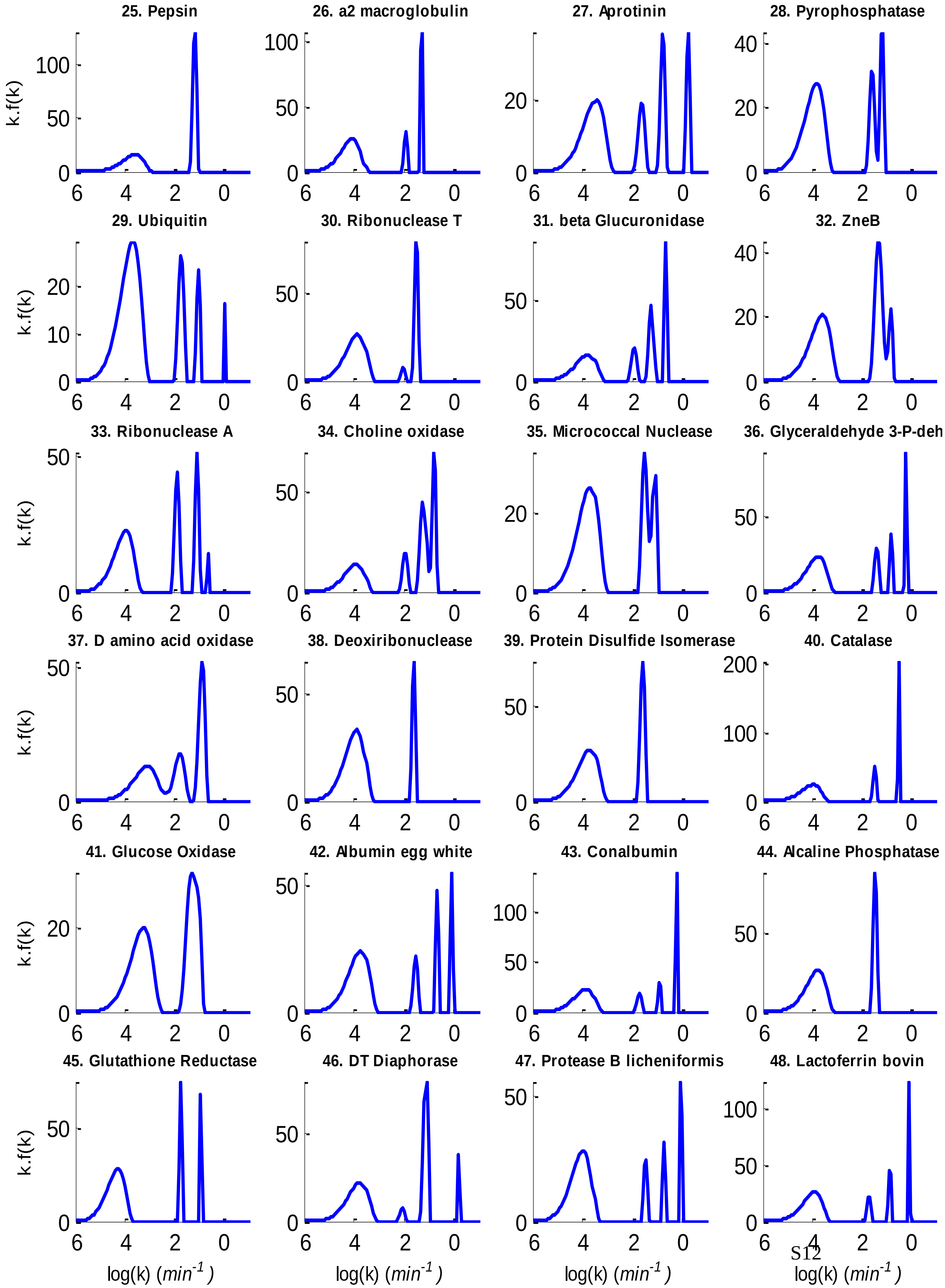

Figure S12c : inverse Laplace transform of the HDX curves (proteins 49-72)

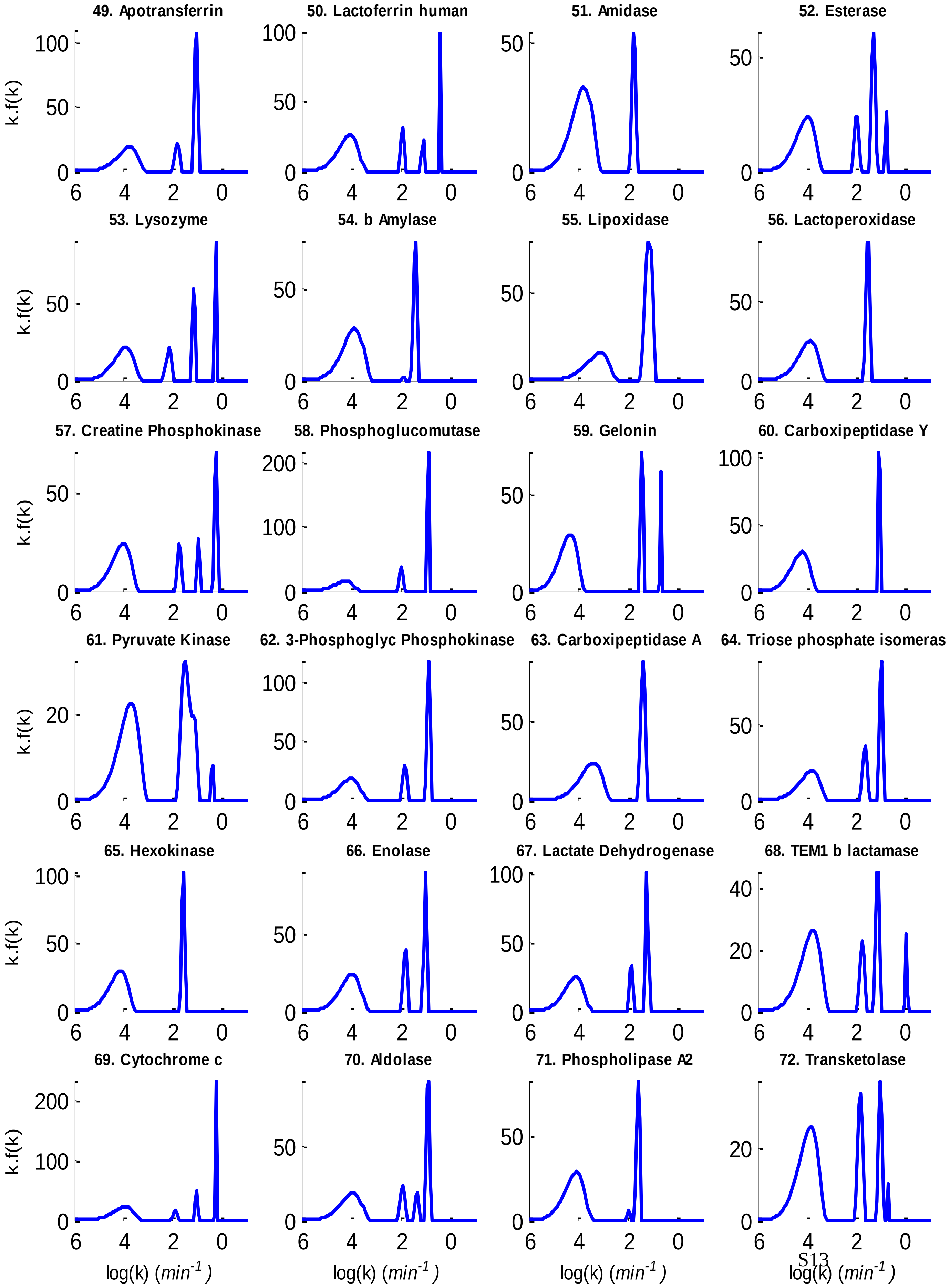

Figure S12d : inverse Laplace transform of the HDX curves (proteins 73-85)

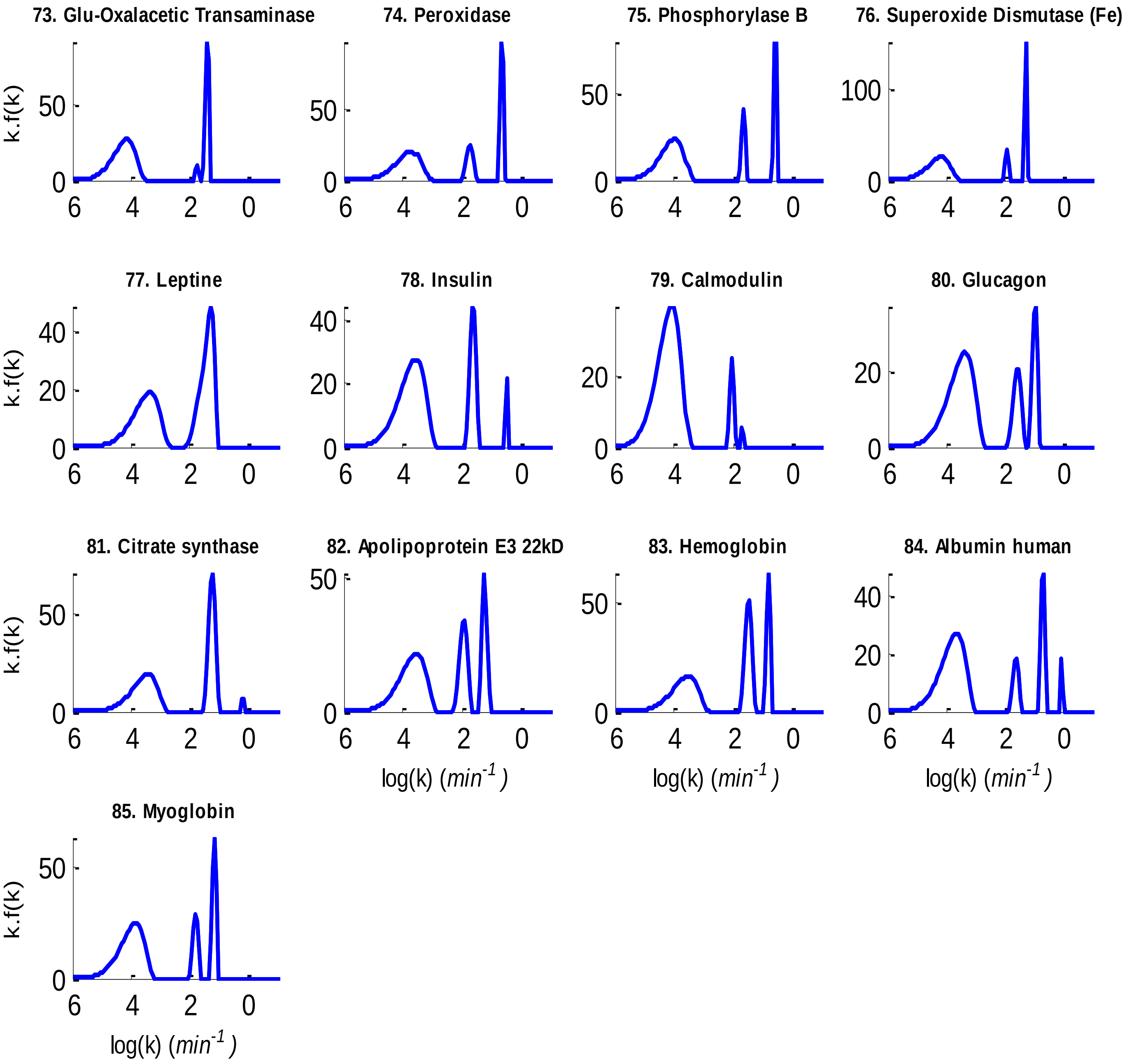

Figure S12: inverse Laplace transform of the HDX curves recorded for the 85 proteins investigated in the present study. It reports k.f(k) as function of $log_{10}(k)$, see Experimental Procedures.

<u>**Figure S13: curve fitting of the 85 proteins by 3 or 4 time constants**</u>

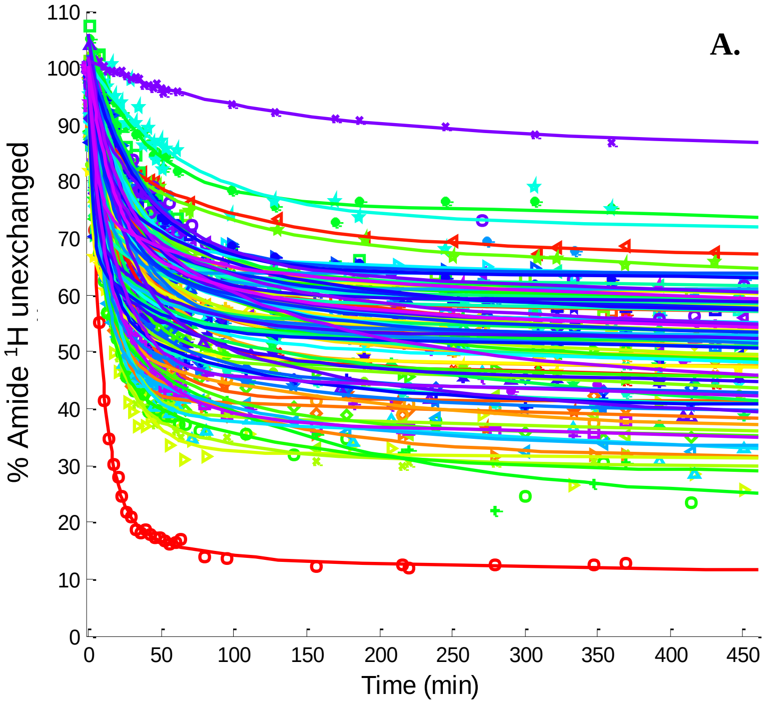

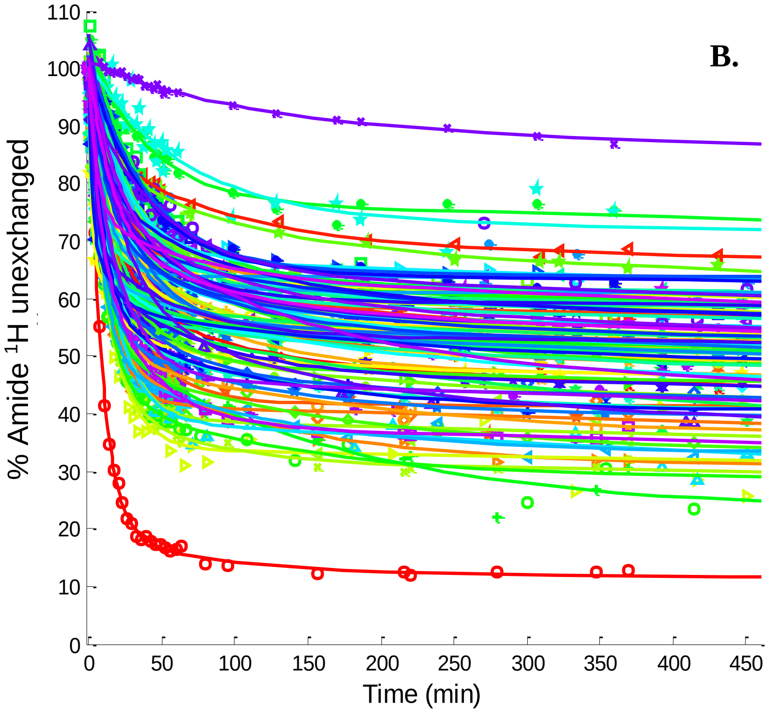

Figure S13: curve fitting for the 85 proteins investigated in the present study by 3 (A.) or 4 (B.) exponential decays characterized by 3 or 4 time constants respectively. The numerical values of the different time constants and of the proportion of each population are reported in Table S3. The fast exchanging protein (red curve at the bottom of the figure) is metallothionein.

Figure S14A: curve fitting results for 3 time constants

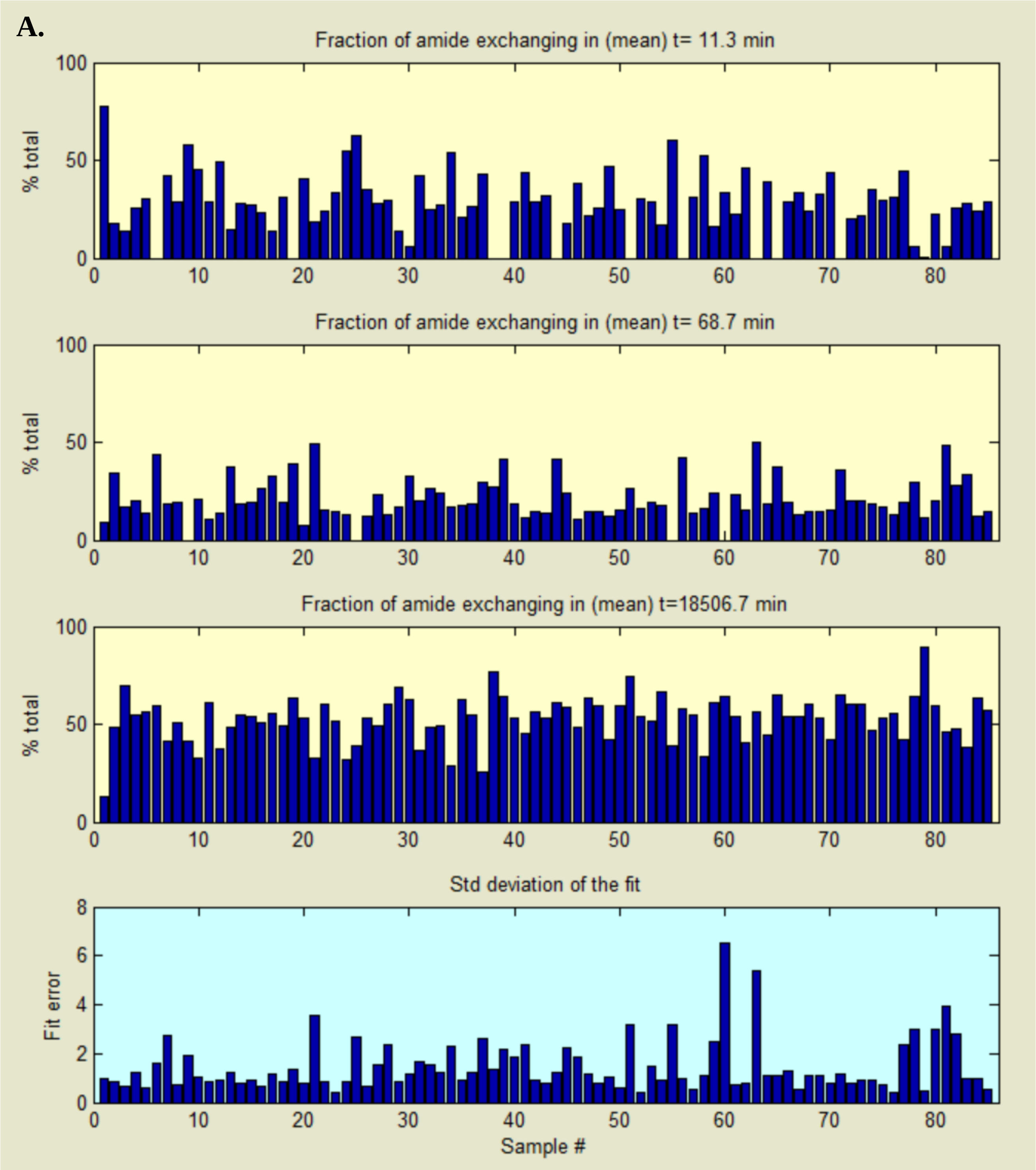

Figure S14B: curve fitting results for 3 time constants, forced to the mean

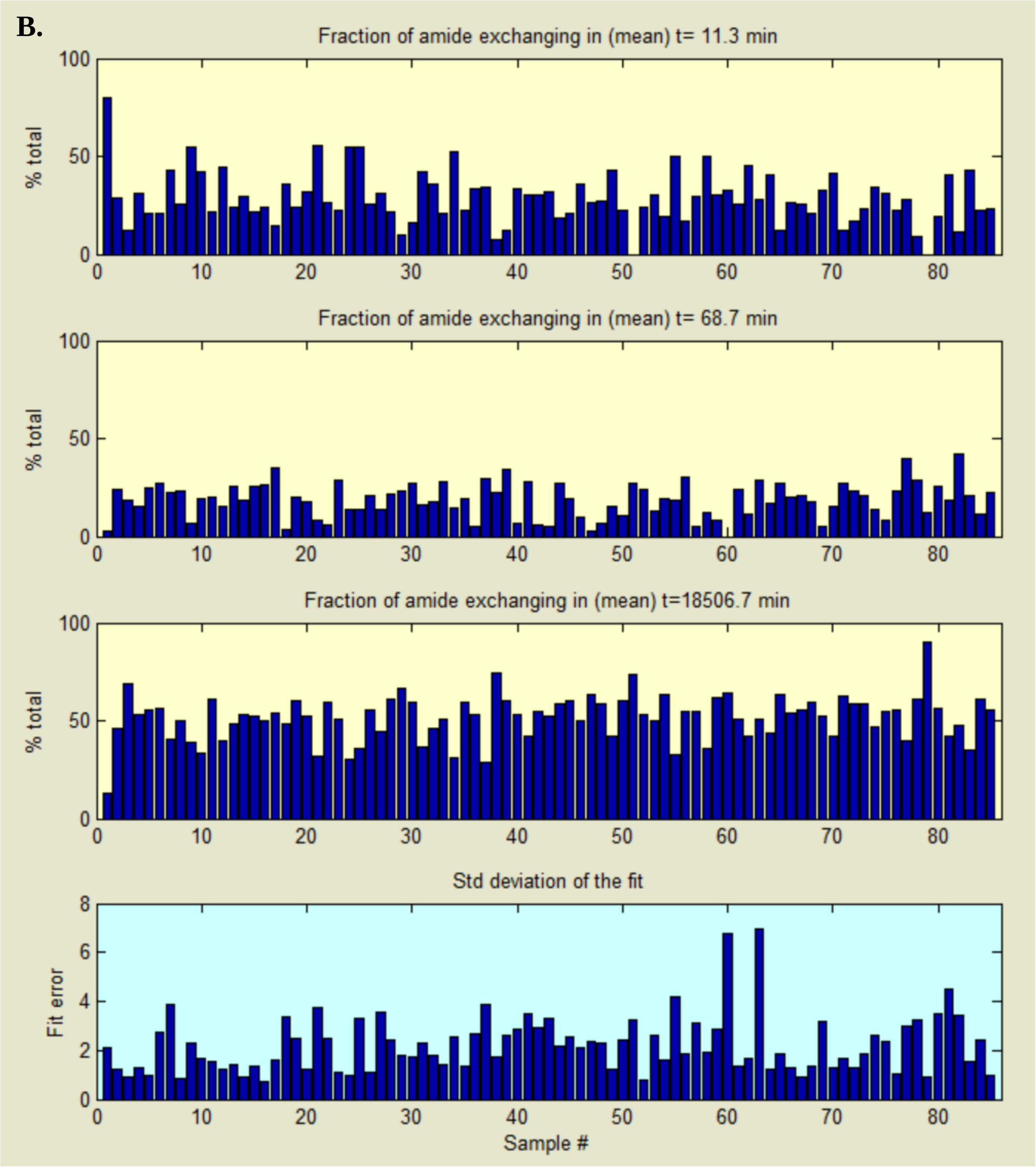

Figure S14C: curve fitting results for 4 time constants

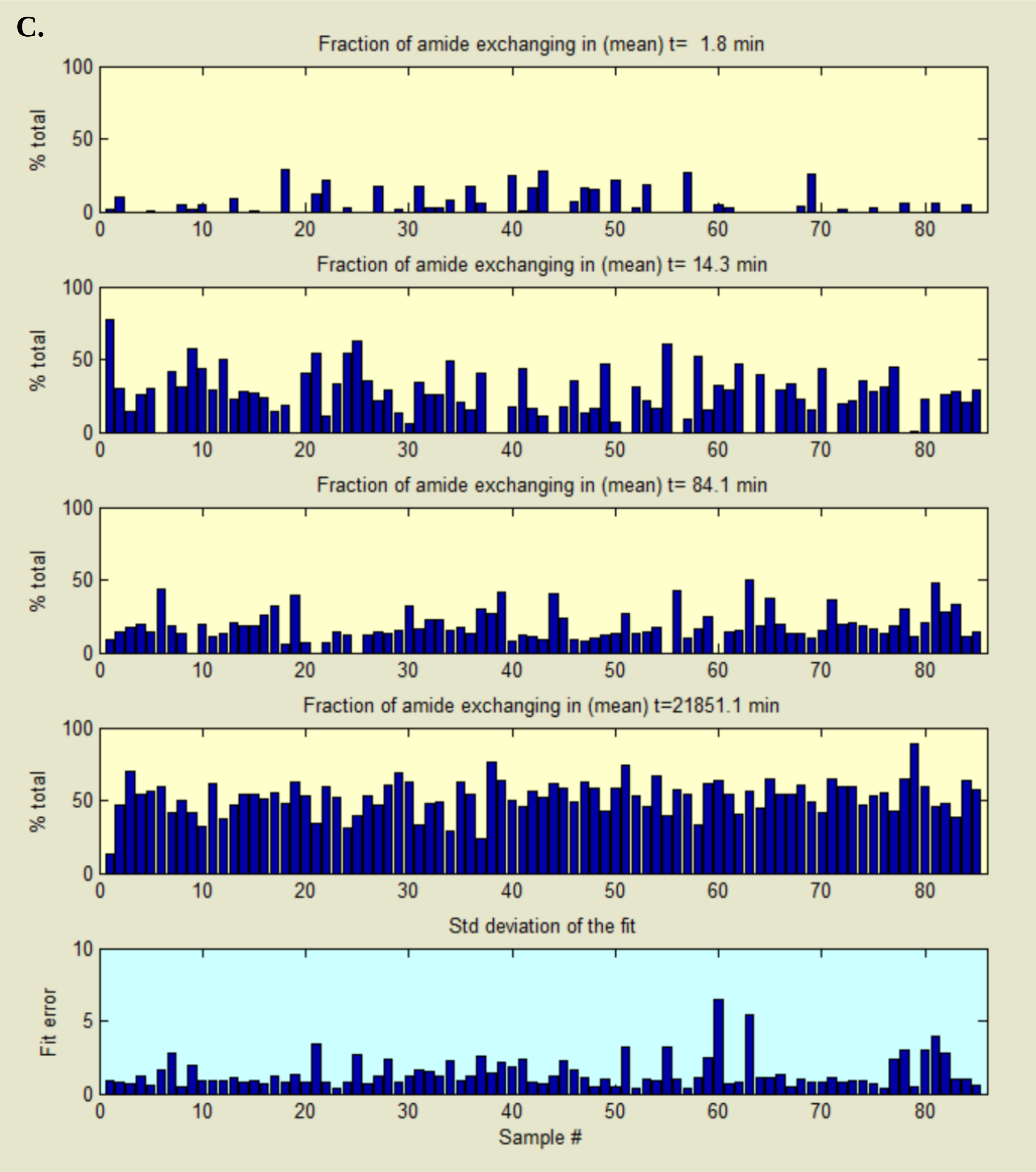

Figure S14: proportion of amide proton present in one of the classes characterized by its time constant. A. for 3 time constants: initial values were 5 min (constrained between 0.01 and 20 min), 46.1 min (constrained between 20 and 200 min) and 6533 min (constrained between 200 and 99,999 min). B. the same fit but constraining the time constants to the mean values obtained in A., except for he first one (5 min). C. for 4 time constants: 1.53 min (between 0.01 and 3 min), 9.8 min (between 3 and 20 min), 46.1 min (between 20 and 200 min) and 6533 min (between 200 and 99,999 min). The corresponding standard deviation of the fit is indicated in the bottom panel of each figure. Sample number corresponds to the numbers provided in Table S1.

Figure S15: correlation between exchange rates and secondary structure content

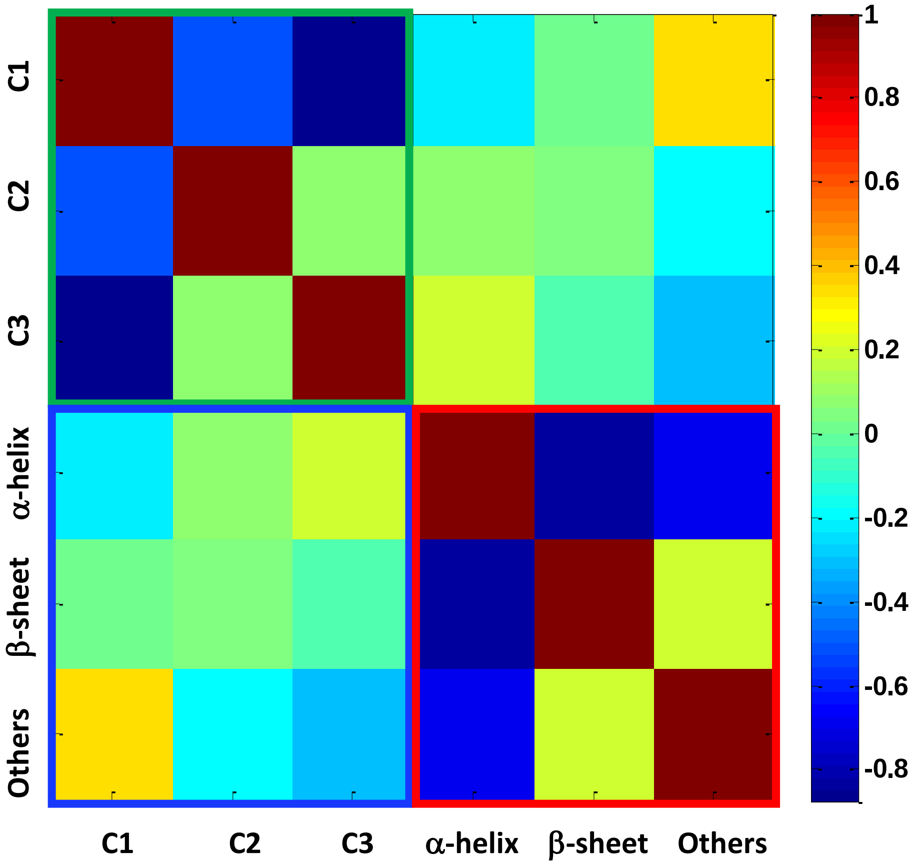

1.0000	-0.5004	-0.8752	-0.2192	0.0328	0.3531	**C1**
-0.5004	1.0000	0.0882	0.0670	0.0490	-0.1865	**C2**
-0.8752	0.0882	1.0000	0.1958	-0.0440	-0.2960	**C3**
-0.2192	0.0670	0.1958	1.0000	-0.8401	-0.6907	**α-helix**
0.0328	0.0490	-0.0440	-0.8401	1.0000	0.1879	**β-sheet**
0.3531	-0.1865	-0.2960	-0.6907	0.1879	1.0000	**Others**
**C1**	**C2**	**C3**	**α-helix**	**β-sheet**	**Others**	

Figure S15: correlation coefficient between the proportions C1, C2 and C3 of amide protons found respectively in each of the three classes characterized by a time constant T1=11.3 min, T2=68.7 min and T3=18506 min. Correlations coefficients are reported graphically (top) and numerically (bottom). The green square indicates the correlations between C1, C2 and C3, the red square between α-helix, β-sheet and "Others" content and the blue square the mixed correlations. In the green square, a strong anti-correlation (R=-0.88) is found between C1 and C3. In the red square, a strong anticorrelation is found between the α-helix and β-sheet content (R=-0.84). In the blue square of interest here, the highest correlation is found between the proportion of the amide protons belonging to the fast-exchanging class C1 and the "Others" content.

Table S1: list of the proteins and their characteristics

Protein #	Protein Name	PDB ID	d3ALPHA;	d3BETA	d3OTHER
1	Metallothionein-2A	4mt2	0	0	100
2	Bowman-Birk proteinase	5j4qr	0	26.76	73.23
3	Trypsin Inhibitor	1ba7	1.65	33.42	64.91
4	Concanavalin A	1l3h	5.06	43.45	51.47
5	Avidin	1vyo	7.03	46.48	46.48
6	SilB-C	2l55	0	48.78	51.21
7	Galactose oxidase	2eie	3.28	39.59	57.12
8	Lectin	1len	3	48.06	48.92
9	Silb-NM2	5a4g	2.22	25	72.77
10	Lysostaphin	4lxc	4.31	42.35	53.33
11	Superoxide Dismutase (Cu Zn)	1q0e	4.6	38.81	56.57
12	IgG human	1hzh	6.25	42.41	51.33
13	Xylanase	2jic	5.26	61.05	33.68
14	Transthyretin Prealbumin	1tta	4.72	48.03	47.24
15	Elastase	1qnj	10	30.41	59.58
16	Pepsinogen	2psg	14.86	36.21	48.91
17	a chymotrypsinogen A	2cga	13.46	32.04	54.48
18	Carbonic anhydrase II	1v9e	15.83	28.95	55.21
19	Carbonic anhydrase I	1hcb	17.69	28.84	53.46
20	Ceruloplsamin	4enz	13.42	34.92	51.64
21	alpha Crystallin B chain	2ygd	13.71	28	58.28
22	beta Lactoglobulin	3npo	16.04	40.74	43.2
23	beta Galactosidase	5a1a	12.13	37.67	50.19
24	Thaumatin II	3aok	12.07	35.74	52.17
25	Pepsin	4pep	15.33	43.25	41.41
26	Alpha-2-Macroglobulin	4acq	14.12	30.18	55.68
27	Aprotinin	4y0y	18.96	24.13	56.89
28	Pyrophosphatase inorganic	1l4o	19.68	28.39	51.91
29	Ubiquitin	2wwz	23.68	31.57	44.73
30	Ribonuclease T	1rls	16.34	27.88	55.76
31	beta Glucuronidase	3lpf	18.49	27.44	54.06
32	ZneB	3lnn	18.94	32.03	49.02
33	Ribonuclease A	1kf5	20.96	33.06	45.96
34	Choline oxidase	4mjw	25.82	20.14	54.02
35	Micrococcal Nuclease	1ey0	24.16	26.84	48.99
36	Glyceraldehyde 3 Phosphate d.	1j0x	31.02	25.52	43.44
37	D amino acid oxidase	1ve9	31.41	26.08	42.5
38	Deoxyribonuclease-1	3dni	28.84	26.15	45
39	Protein Disulfide Isomerase	4el1	28.83	19.19	51.97
40	Catalase	3rgp	32.46	16.63	50.9
41	Glucose Oxidase	1cf3	34.13	19.21	46.65
42	Albumin egg white	1ova	29.14	28.88	41.96
43	Conalbumin	1ovt	32.79	17.63	49.56
44	Alcaline Phosphatase	1y6v	31.06	18.15	50.77

45	Glutathione Reductase	3djg	33.96	23.48	42.55
46	DT Diaphorase	1d4a	37.36	11.35	51.28
47	Protease Bacillus licheniformis	3unx	29.56	17.88	52.55
48	Lactoferrin bovin	1blf	31.78	17.56	50.65
49	Apotransferrin	4h0w	34.46	18.11	47.42
50	Lactoferrin human	1cb6	36.17	18.81	45
51	Amidase	2uxy	32.55	21.7	45.74
52	Esterase	1k4y	33.52	13.85	52.62
53	Lysozyme	4lzt	40.31	6.2	53.48
54	b Amylase	1fa2	38.35	11.44	50.2
55	Lipoxidase	1f8n	40.64	13.11	46.24
56	Lactoperoxidase	6a4y	37.47	5.71	56.8
57	Creatine Phosphokinase	1u6r	38.55	14.21	47.23
58	Phosphoglucomutase	5epc	34.99	23.49	41.5
59	Gelonin	3ktz	37.05	21.11	41.83
60	Carboxipeptidase Y	1ysc	38.47	14.25	47.26
61	Pyruvate Kinase	1a49	38.46	18.86	42.66
62	3 Phosphoglycerate kinase	1qpg	41.2	16.62	42.16
63	Carboxipeptidase A	2ctb	38.76	16.28	44.95
64	Triose phosphate isomerase2(8)	1ypi	43.31	16.19	40.48
65	Hexokinase	1ig8	42.18	16.04	41.76
66	Enolase	1ebh	45.29	16.97	37.72
67	Lactate Dehydrogenase	3h3f	44.29	19.75	35.95
68	Beta-Lactamase TEM	1xpb	44.86	17.11	38.02
69	Cytochrome c	1hrc	40.95	0	59.04
70	Aldolase	1zah	44.97	13.77	41.25
71	Phospholipase A2	2osh	47.89	6.72	45.37
72	Transketolase	2r8o	46.86	13.6	39.53
73	Glutamate oxaloacetate transaminase-1	5toq	45.63	13.34	41.01
74	Peroxidase	1hch	50	1.96	48.03
75	Glycogen phosphorylase-B	1axr	50.11	14.25	35.62
76	Superoxide Dismutase (Fe)	1isa	52.34	10.93	36.71
77	Leptine	1ax8	60.95	0	39.04
78	Insulin	3w7y	50.98	5.88	43.13
79	Calmodulin	1prw	57.71	2.68	39.59
80	Glucagon	1nau	60.71	0	39.28
81	Citrate synthase	3enj	61.78	3.43	34.78
82	Apolipoprotein E3	1h7i	62.3	0	37.69
83	Hemoglobin	2qsp	77.09	0	22.9
84	Serum albumin	1n5u	71.96	0	28.03
85	Myoglobin	1wla	73.85	0	26.14

Table S1: List of the 85 proteins used in this study. The PDB entry number of and DSSP-derived fraction of α-helix, β-sheet and Others structures are provided. Proteins have been sorted in order of increasing α-helix content. The color code is designed to facilitate reading. It is the same as in Tables S2 and S3. Details about the proteins (PDB ID, structure resolution, CATH superfamily etc.) can be found in (De Meutter and Goormaghtigh 2020)

Table S2: time constants obtained by inverse Laplace transform for all proteins

Protein	T1	T2	T3	T4
Metallothionein-2A	1892.46	46.11	8.39	
Bowman-Birk proteinase	4798.77	24.83	3.87	
Trypsin Inhibitor	7628.92	53.78	7.19	
Concanavalin A	5603.76	28.99	8.39	
Avidin	6533.02	46.11	15.61	
SilB-C	4798.77	24.83		
Galactose oxidase	19308.04	21.27		
Lectin	6533.02	62.89	15.61	1.12
Silb-NM2	3519.27	11.45	0.01	
Lysostaphin	5603.76	73.48	8.39	
Superoxide Dismutase (Cu Zn)	12135.08	73.48	21.27	
IgG human	6533.02	100.00	9.81	
Xylanase	8914.37	46.11	21.27	2.43
Transthyretin Prealbumin	7628.92	46.11	9.81	
Elastase	4798.77	53.78	11.45	
Pepsinogen	7628.92	46.11	9.81	
a chymotrypsinogen A	7628.92	73.48	28.99	
Carbonic anhydrase II	5603.76	24.83	9.81	1.12
Carbonic anhydrase I	6533.02	21.27		
Ceruloplsamin	6533.02	85.72	13.37	
alpha Crystallin B chain	2578.37	13.37	0.60	
beta Lactoglobulin	6533.02	39.49	15.61	1.79
beta Galactosidase	6533.02	100.00	24.83	15.61
Thaumatin II	4798.77	53.78	11.45	
Pepsin	3519.27	13.37		
Alpha-2-Macroglobulin	10394.13	73.48	15.61	
Aprotinin	2578.37	39.49	6.17	0.52
Pyrophosphatase inorganic	6533.02	39.49	13.37	
Ubiquitin	4110.84	53.78	9.81	0.83
Ribonuclease T	6533.02	100.00	28.99	
beta Glucuronidase	5603.76	73.48	15.61	4.52
ZneB	3519.27	21.27	6.17	
Ribonuclease A	8914.37	73.48	11.45	3.87
Choline oxidase	6533.02	85.72	15.61	6.17
Micrococcal Nuclease	4798.77	28.99	11.45	
Glyceraldehyde 3 Phosphate d.	4798.77	24.83	6.17	1.53
D amino acid oxidase	1019.31	53.78	7.19	
Deoxyribonuclease-1	6533.02	33.82		
Protein Disulfide Isomerase	4798.77	33.82		
Catalase	8914.37	28.99	2.84	
Glucose Oxidase	1621.60	18.22		
Albumin egg white	4798.77	28.99	4.52	1.12
Conalbumin	6533.02	46.11	8.39	1.53
Alcaline Phosphatase	5603.76	28.99		
Glutathione Reductase	16536.72	53.78	8.39	
DT Diaphorase	4798.77	100.00	11.45	0.60
Protease Bacillus licheniformis	8914.37	24.83	5.28	1.12
Lactoferrin bovin	7628.92	53.78	7.19	1.12
Apotransferrin	4798.77	62.89	9.81	
Lactoferrin human	8914.37	73.48	11.45	2.43
Amidase	5603.76	53.78		

Esterase	8914.37	100.00	18.22	5.28
Lysozyme	6533.02	136.28	13.37	1.53
b Amylase	6533.02	73.48	21.27	
Lipoxidase	1189.78	13.37	0.01	
Lactoperoxidase	6533.02	28.99		
Creatine Phosphokinase	8914.37	53.78	8.39	1.53
Phosphoglucomutase	14167.11	85.72	7.19	
Gelonin	19308.04	24.83	4.52	
Carboxipeptidase Y	14167.11	11.45	0.01	
Pyruvate Kinase	4110.84	28.99	13.37	2.09
3 Phosphoglycerate kinase	8914.37	62.89	7.19	
Carboxipeptidase A	2209.22	21.27		
Triose phosphate isomerase2(8)	5603.76	39.49	8.39	
Hexokinase	12135.08	33.82		
Enolase	7628.92	53.78	9.81	
Lactate Dehydrogenase	10394.13	62.89	15.61	
Beta-Lactamase TEM	4798.77	53.78	11.45	0.83
Cytochrome c	7628.92	73.48	9.81	1.53
Aldolase	7628.92	73.48	18.22	7.19
Phospholipase A2	10394.13	85.72	33.82	
Transketolase	6533.02	62.89	9.81	4.52
Glutamate oxaloacetate transaminase-1	12135.08	53.78	24.83	
Peroxidase	4798.77	46.11	4.52	
Glycogen phosphorylase-B	8914.37	39.49	3.87	
Superoxide Dismutase (Fe)	14167.11	85.72	18.22	
Leptine	2209.22	18.22		
Insulin	3519.27	39.49	2.84	
Calmodulin	10394.13	100.00	46.11	
Glucagon	2209.22	39.49	8.39	
Citrate synthase	2209.22	15.61	1.31	
Apolipoprotein E3	3519.27	73.48	15.61	
Hemoglobin	3012.20	24.83	6.17	
Serum albumin	4110.84	39.49	4.52	1.12
Myoglobin	6533.02	62.89	13.37	
**median**	**6533.00**	**46.10**	**9.80**	**1.53**

Table S2: time constants (in min) obtained by inverse Laplace transform for all proteins. The time constants T1, T2, T3 and T4 were obtained from the position of the maxima found in Figure S12. The values reported in this table is 10^k, where k is the position of the maxima found in Figure S12. The color code is designed to facilitate reading. It is the same as in Tables S1 and S3.

Table S3: time constants obtained by curve fitting for all proteins

	Fitting with 3 time constants							Fitting with 4 time constants									
	C1	T1	C2	T2	C3	T3	stddev	C0	T0	C1	T1	C2	T2	C3	T3	stddev	diff stddev
Metallothionein-2A	77.4	8.6	9.1	54.1	13.0	4000.9	1.0	1.4	0.0	77.1	8.8	8.5	56.9	13.0	4009.7	0.9	-0.01
Bowman-Birk proteinase	17.6	5.5	34.2	39.1	48.5	7538.1	0.9	9.6	3.0	30.0	20.0	13.9	102.3	47.0	9364.6	0.8	-0.05
Trypsin Inhibitor	13.9	10.0	17.0	84.5	69.6	13028.2	0.6	0.0	3.0	14.0	10.1	16.9	85.0	69.6	13050.9	0.6	0.00
Concanavalin A	25.4	10.1	20.0	43.3	54.6	12134.1	1.2	0.0	2.8	25.4	10.1	20.0	43.3	54.6	12134.1	1.2	0.00
Avidin	30.1	19.2	13.8	116.4	56.1	13082.8	0.6	0.1	0.0	30.1	19.3	13.8	117.4	56.0	13149.1	0.6	0.00
SilB-C	0.0	19.2	43.4	30.6	59.3	11433.7	1.6	0.0	0.2	0.0	19.8	43.4	30.6	59.3	11433.7	1.6	0.00
Galactose oxidase	42.0	20.0	18.3	33.5	41.6	99999.0	2.8	0.0	3.0	42.0	20.0	18.3	33.5	41.6	99999.0	2.8	0.00
Lectin	28.9	13.3	18.9	78.0	50.6	11328.5	0.7	5.3	1.3	31.4	18.9	13.6	116.9	49.8	13019.1	0.5	-0.22
Silb-NM2	57.6	12.7	0.0	40.5	41.6	5730.2	1.9	1.5	0.0	57.0	12.9	0.0	23.5	41.5	5776.5	1.9	-0.01
Lysostaphin	45.1	9.2	20.5	95.7	32.4	13969.2	1.0	4.7	0.1	44.0	10.3	19.6	111.3	31.8	17378.1	0.9	-0.16
Superoxide Dismutase (Cu Zn)	29.1	20.0	10.9	85.9	61.4	25165.4	0.9	0.0	2.8	29.1	20.0	10.9	85.9	61.4	25165.5	0.9	0.00
IgG human	49.6	11.9	13.5	171.3	37.0	18663.1	0.9	0.0	0.5	49.6	11.9	13.5	171.3	37.0	18663.2	0.9	0.00
Xylanase	14.3	3.8	37.0	44.5	48.3	27767.8	1.2	9.4	2.0	22.5	20.0	21.0	82.3	47.1	60050.7	1.1	-0.11
Transthyretin Prealbumin	28.1	11.2	18.5	56.6	54.6	12044.0	0.8	0.0	2.2	28.1	11.2	18.5	56.6	54.6	12044.0	0.8	0.00
Elastase	27.1	13.5	18.9	85.5	53.7	8124.2	0.9	0.6	0.0	27.1	13.9	18.6	87.4	53.7	8177.5	0.9	0.00
Pepsinogen	23.6	12.3	26.2	61.2	50.9	12967.3	0.7	0.0	3.0	23.6	12.3	26.2	61.2	50.9	12967.3	0.7	0.00
a chymotrypsinogen A	14.1	20.0	32.5	55.3	55.4	13995.8	1.2	0.0	2.4	14.1	20.0	32.5	55.3	55.4	13995.8	1.2	0.00
Carbonic anhydrase II	31.0	1.4	19.6	23.5	49.4	9933.6	0.9	28.5	1.2	18.1	15.0	5.6	112.9	47.8	14503.8	0.7	-0.13
Carbonic anhydrase I	0.0	19.4	39.2	25.5	63.1	9963.7	1.3	0.0	1.1	0.0	19.6	39.2	25.5	63.1	9963.7	1.3	0.00
Ceruloplsamin	40.5	17.2	7.3	200.0	52.9	11585.0	0.8	0.0	3.0	40.5	17.2	7.3	200.0	52.9	11585.0	0.8	0.00
alpha Crystallin B chain	18.3	1.5	49.4	20.0	32.4	12135.4	3.6	11.9	0.9	54.3	16.0	0.0	20.0	33.8	7801.5	3.4	-0.15
beta Lactoglobulin	23.9	2.0	15.3	31.9	60.6	10627.3	0.8	21.3	1.7	11.4	15.4	7.3	70.2	59.9	12072.5	0.8	-0.06
beta Galactosidase	33.7	20.0	14.7	103.7	51.9	9187.6	0.4	0.0	1.5	33.7	20.0	14.7	103.7	51.9	9187.6	0.4	0.00
Thaumatin II	54.4	11.1	13.0	60.5	31.6	8042.5	0.9	2.3	0.1	54.3	11.7	11.8	65.2	31.6	8089.2	0.8	-0.05
Pepsin	62.7	15.9	0.0	20.0	39.0	5786.7	2.7	0.0	2.9	62.7	15.9	0.0	20.7	39.0	5786.7	2.7	0.00
Alpha-2-Macroglobulin	34.9	18.8	12.1	172.5	53.3	35908.3	0.6	0.0	0.5	34.9	18.8	12.1	172.5	53.3	35909.6	0.6	0.00
Aprotinin	27.7	1.4	23.1	28.0	48.8	4017.8	1.5	17.2	0.7	21.8	7.7	13.9	64.2	47.1	4770.8	1.2	-0.32
Pyrophosphatase inorganic	29.4	18.4	12.9	111.1	60.5	19735.4	2.4	0.0	0.0	29.4	18.4	12.9	111.1	60.5	19735.4	2.4	0.00
Ubiquitin	13.4	11.3	16.9	81.3	69.1	6957.1	0.9	2.0	1.0	13.5	14.3	15.6	90.0	68.9	7052.9	0.8	-0.02
Ribonuclease T	6.0	20.0	32.4	36.7	62.4	9015.1	1.2	0.0	2.9	6.0	20.0	32.4	36.7	62.4	9015.1	1.2	0.00
beta Glucuronidase	42.5	7.4	20.5	70.5	36.8	11065.4	1.7	16.9	3.0	34.6	13.9	16.3	175.1	33.0	99999.0	1.6	-0.09
ZneB	24.5	9.1	26.3	33.9	48.4	7368.5	1.5	2.4	3.0	25.7	11.0	23.0	36.7	48.4	7400.9	1.5	0.00
Ribonuclease A	26.8	12.6	24.0	116.4	49.1	25982.3	1.2	2.9	3.0	25.8	14.8	22.9	127.0	48.7	30421.3	1.2	-0.01
Choline oxidase	54.3	9.8	16.7	107.2	29.1	99999.0	2.3	8.1	3.0	48.8	11.7	15.1	122.2	28.9	99999.0	2.3	-0.02
Micrococcal Nuclease	20.8	14.8	17.4	45.1	62.3	8313.1	0.9	0.0	3.0	20.8	14.8	17.4	45.1	62.3	8313.1	0.9	0.00

Glyceraldehyde3Phosphate d	26.7	2.4	18.8	26.6	54.3	9569.0	1.2	17.7	1.5	15.7	7.4	12.8	39.2	53.9	10356.0	1.2	-0.07
D amino acid oxidase	42.9	8.0	29.4	141.6	25.3	8318.5	2.6	5.7	0.0	40.6	9.1	29.8	161.7	23.8	13094.8	2.5	-0.09
Deoxyribonuclease-1	0.0	19.1	26.9	40.3	76.8	11391.1	1.4	0.0	0.7	0.0	19.3	26.9	40.3	76.8	11391.1	1.4	0.00
Protein Disulfide Isomerase	0.0	18.0	41.5	39.7	63.9	8419.2	2.2	0.0	3.0	0.0	16.3	41.5	39.7	63.9	8419.2	2.2	0.00
Catalase	28.7	3.4	18.6	38.3	53.1	19518.0	1.8	24.9	2.9	17.1	20.0	8.2	186.3	50.4	99999.0	1.8	-0.05
Glucose Oxidase	43.5	16.4	11.8	200.0	45.4	3754.2	2.4	0.1	3.0	43.5	16.5	11.7	200.0	45.5	3750.8	2.4	0.00
Albumin egg white	28.7	2.5	14.3	34.2	56.4	8429.4	0.9	16.6	1.3	16.6	6.1	10.8	50.8	56.0	9071.3	0.8	-0.11
Conalbumin	32.2	2.3	14.1	39.2	53.5	9101.6	0.8	27.5	1.8	11.2	12.1	8.8	83.5	52.5	10787.5	0.7	-0.11
Alcaline Phosphatase	0.0	17.0	41.0	32.7	61.2	12687.7	1.2	0.0	0.1	0.0	20.0	41.0	32.7	61.2	12687.7	1.2	0.00
Glutathione Reductase	17.8	8.7	23.8	63.5	58.8	99999.0	2.2	0.0	2.4	17.8	8.7	23.8	63.5	58.8	99999.0	2.2	0.00
DT Diaphorase	38.5	10.8	10.3	200.0	48.2	19140.6	1.9	7.1	0.5	35.3	12.7	8.9	200.0	48.7	16706.3	1.7	-0.21
Protease Bacillus licheniformis	22.0	1.8	14.5	28.5	63.3	16994.4	1.2	16.6	1.2	13.1	9.6	7.9	65.0	62.4	22071.1	1.1	-0.09
Lactoferrin bovin	25.5	2.9	14.2	42.2	59.4	11751.7	0.8	15.1	1.3	16.6	8.6	9.9	83.9	58.5	14432.3	0.5	-0.30
Apotransferrin	46.7	11.5	12.5	106.6	42.3	11282.4	1.1	0.0	2.5	46.7	11.5	12.5	106.6	42.3	11282.4	1.1	0.00
Lactoferrin human	24.9	3.2	15.1	73.4	59.7	13338.1	0.6	21.3	2.5	7.2	15.1	12.6	110.0	58.8	16034.2	0.5	-0.08
Amidase	0.0	3.2	26.6	64.0	74.2	14484.6	3.2	0.0	3.0	0.0	16.8	26.6	64.0	74.2	14484.7	3.2	0.00
Esterase	30.0	15.6	16.0	96.5	53.9	11472.5	0.4	2.8	3.0	31.3	19.2	13.1	132.6	53.1	13086.9	0.4	-0.04
Lysozyme	28.6	3.7	19.5	47.5	51.2	7606.5	1.5	18.3	1.8	21.7	13.8	14.5	200.0	45.8	28396.0	1.0	-0.53
b Amylase	16.8	20.0	17.3	33.5	66.6	9036.6	0.9	0.0	0.2	16.8	20.0	17.3	33.5	66.6	9036.6	0.9	0.00
Lipoxidase	60.1	16.2	0.0	20.0	39.0	2781.6	3.2	0.0	2.4	60.1	16.2	0.0	88.3	39.0	2781.6	3.2	0.00
Lactoperoxidase	0.0	16.5	42.4	35.6	57.7	10117.8	1.0	0.0	3.0	0.0	17.7	42.4	35.6	57.7	10117.8	1.0	0.00
Creatine Phosphokinase	30.8	2.1	14.0	42.3	54.9	11578.0	0.5	26.5	1.7	8.9	10.6	10.2	67.8	54.3	13132.3	0.4	-0.13
Phosphoglucomutase	52.3	8.7	16.4	117.4	33.2	99999.0	1.1	0.0	2.5	52.3	8.7	16.4	117.4	33.2	99999.0	1.1	0.00
Gelonin	15.8	5.7	24.4	31.0	61.2	99999.0	2.5	0.0	2.9	15.8	5.7	24.4	31.0	61.2	99999.0	2.5	0.00
Carboxipeptidase Y	33.7	11.6	0.0	20.7	63.9	99999.0	6.5	4.5	0.0	31.7	12.3	0.0	31.5	63.8	99999.0	6.5	-0.03
Pyruvate Kinase	22.8	13.3	22.9	46.5	54.0	6947.9	0.7	2.7	2.6	28.9	18.4	14.6	61.1	53.8	7061.4	0.7	-0.02
3 Phosphoglycerate kinase	46.4	8.2	15.0	90.4	40.6	22287.9	0.8	0.0	1.7	46.4	8.2	15.0	90.4	40.6	22287.9	0.8	0.00
Carboxipeptidase A	0.0	19.8	49.7	25.5	56.3	5081.0	5.4	0.0	1.7	0.0	19.2	49.7	25.5	56.3	5081.0	5.4	0.00
Triose phosphate isomerase2(8)	39.0	10.0	18.8	59.0	44.6	11779.9	1.1	0.0	3.0	39.0	10.0	18.8	59.0	44.6	11779.9	1.1	0.00
Hexokinase	0.0	16.1	37.1	40.0	64.9	25862.7	1.1	0.0	3.0	0.0	19.4	37.1	40.0	64.9	25862.7	1.1	0.00
Enolase	28.4	11.1	19.1	82.2	53.8	16921.7	1.3	0.0	2.9	28.4	11.1	19.1	82.2	53.8	16921.7	1.3	0.00
Lactate Dehydrogenase	33.3	17.7	13.1	124.6	54.0	30551.8	0.5	0.0	0.0	33.3	17.7	13.1	124.6	54.0	30551.8	0.5	0.00
Beta-Lactamase TEM	23.9	11.6	14.3	81.2	60.3	9918.7	1.1	4.2	0.9	23.0	14.3	12.7	92.5	60.1	10111.8	1.0	-0.09
Cytochrome c	32.3	2.4	14.5	38.5	53.0	10895.4	1.1	25.8	1.7	15.6	12.6	9.7	200.0	49.1	38435.1	0.8	-0.30
Aldolase	43.8	9.6	15.4	93.4	42.0	13310.4	0.8	0.0	2.9	43.8	9.6	15.4	93.4	42.0	13310.4	0.8	0.00
Phospholipase A2	0.0	18.1	35.9	39.1	64.7	16420.2	1.2	0.0	2.4	0.0	18.7	35.9	39.1	64.7	16420.2	1.2	0.00
Transketolase	19.9	10.3	20.0	85.0	60.0	9508.2	0.8	1.9	3.0	19.0	11.8	19.3	89.7	59.9	9647.6	0.8	0.00
Glutamate oxaloacetate transaminase-1	21.4	20.0	20.1	42.5	59.9	25623.6	0.9	0.0	2.6	21.4	20.0	20.1	42.5	59.9	25623.6	0.9	0.00
Peroxidase	35.1	5.0	18.1	68.0	47.1	9152.7	0.9	0.0	2.8	35.1	5.0	18.1	68.0	47.1	9152.7	0.9	0.00
Glycogen phosphorylase-B	29.5	3.8	16.6	53.1	53.5	21801.0	0.7	2.3	0.5	28.0	4.2	16.2	55.1	53.5	22264.5	0.7	-0.02

Superoxide Dismutase (Fe)	31.2	20.0	13.1	102.0	55.7	22028.9	0.4	0.0	3.0	31.2	20.0	13.1	102.0	55.7	22028.9	0.4	0.00
Leptine	44.8	20.0	18.9	121.7	42.3	5806.0	2.3	0.0	0.0	44.8	20.0	18.9	121.7	42.3	5806.0	2.3	0.00
Insulin	5.9	3.5	29.7	50.0	64.4	7334.1	3.0	5.8	3.0	0.0	12.8	29.9	49.5	64.5	7316.2	3.0	0.00
Calmodulin	0.6	20.0	11.3	114.3	89.5	13824.0	0.5	0.0	1.8	0.6	20.0	11.3	114.3	89.5	13824.0	0.5	0.00
Glucagon	22.4	10.1	20.2	71.9	59.4	5679.5	3.0	0.0	3.0	22.4	10.1	20.2	71.9	59.4	5679.5	3.0	0.00
Citrate synthase	6.0	2.7	48.1	21.4	46.1	5402.6	4.0	6.0	2.7	0.0	19.4	48.1	21.4	46.1	5402.6	4.0	0.00
Apolipoprotein E3	25.6	17.8	28.3	128.4	47.8	7761.0	2.8	0.0	0.0	25.6	17.8	28.3	128.4	47.8	7761.0	2.8	0.00
Hemoglobin	28.1	7.0	33.5	33.9	38.1	5351.9	1.0	0.0	2.4	28.1	7.0	33.5	33.9	38.1	5351.9	1.0	0.00
Serum albumin	23.9	4.9	11.9	59.4	63.8	6496.9	1.0	4.9	1.4	20.5	6.2	11.0	67.7	63.6	6604.5	1.0	-0.02
Myoglobin	28.9	15.6	14.4	89.6	56.9	9955.7	0.6	0.0	0.0	28.9	15.6	14.4	89.6	56.9	9955.7	0.6	0.00
**Median values**	**28.1**	**11.2**	**18.5**	**55.3**	**53.9**	**11391.0**	**1.1**	**0**	**2**	**25.6**	**14.8**	**15.4**	**71.9**	**53.8**	**12.1**	**1.0**	**0**
	**C1**	**T1**	**C2**	**T2**	**C3**	**T3**	**stddev**	**C0**	**T0**	**C1**	**T1**	**C2**	**T2**	**C3**	**T3**	**stddev**	**diff stddev**

Table S3: Results of the curve fitting of the HDX curves for the 85 proteins. A first curve fitting was done with 3 time constants T1, T2 and T3. Time constants are in minutes. The corresponding proportions of the amide protons are C1, C2 and C3 respectively. Values of the C_i are in %. The standard deviation of the fit is indicated under "stddev". A second first curve fitting was done with 4 time constants T0, T1, T2 and T3. The corresponding proportions of the amide groups are C0, C1, C2 and C3 respectively. The standard deviation of the fit is indicated under "stddev". The difference between the standard deviations obtained for the two curve fittings appears in the last column. The median value of each column appears in the last line of the table.

Curve fitting initial parameters and constrains applied were as follows:

		initial value	minimum	maximum
<u>**3 time constants**</u> **(min)**	**T1**	5	0.01	20
	**T2**	46.1	20	200
	**T3**	6533	200	99999
<u>**4 time constants**</u> **(min)**	**T0**	1.53	0.01	3
	**T1**	9.8	3	20
	**T2**	46.1	20	200
	**T3**	6533	200	99999

All time constant values are given in min.

The color code is designed to facilitate reading. It is the same as in Tables S1 and S2.

References

Barth A (2007) Infrared spectroscopy of proteins. Biochim Biophys Acta 1767:1073–1101

Chirgadze YN, Fedorov O V, Trushina NP (1975) Estimation of amino acid residue side-chain absorption in the infrared spectra of protein solutions in heavy water. Biopolymers 14:679–694

De Meutter J, Goormaghtigh E (2021) Evaluation of protein secondary structure from FTIR spectra improved after partial deuteration. Eur Biophys J. https://doi.org/10.1007/s00249-021-01502-y

De Meutter J, Goormaghtigh E (2020) A convenient protein library for spectroscopic calibrations. Comput Struct Biotechnol J 18:1864–1876. https://doi.org/10.1016/J.CSBJ.2020.07.001

Goormaghtigh E (2009) FTIR Data Processing and Analysis Tools. In: Barth A, P.I.Haris (eds) Adv. Biomed. Spectrosc. (Biological and Biomedical Infrared Spectroscopy). IOS Press, pp 104–128

Goormaghtigh E, De-Jongh HH, Ruysschaert JM (1996) Relevance of protein thin films prepared for attenuated total reflection Fourier transform infrared spectroscopy: significance of the pH. ApplSpectrosc 50:1519–1527

Goormaghtigh E, Vigneron L, Scarborough GA, Ruysschaert JM (1994) Tertiary conformational changes of the Neurospora crassa plasma membrane H(+)-ATPase monitored by hydrogen/deuterium exchange kinetics. A Fourier transformed infrared spectroscopy approach. JBiolChem 269:27409–27413

Hollecker M, Vincent M, Gallay J, et al (2002) Insight into the factors influencing the backbone dynamics of three homologous proteins, dendrotoxins I and K, and BPTI: FTIR and time-resolved fluorescence investigations. Biochemistry 41:15267–15276

Provencher SW (1982) CONTIN: A general purpose constrained regularization program for inverting noisy linear algebraic and integral equations. Comput Phys Commun 27:229–242

Provencher SW, Dovi VG (1979) Direct analysis of continuous relaxation spectra. JBiochemBiophysMethods 1:313–318

Rahmelow K, Hubner W, Ackermann T (1998) Infrared absorbances of protein side chains. AnalBiochem 257:1–11

Raussens V, Narayanaswami V, Goormaghtigh E, et al (1996) Hydrogen/deuterium exchange kinetics of apolipophorin-III in lipid-free and phospholipid-bound states. An analysis by Fourier transform infrared spectroscopy. JBiolChem 271:23089–23095

Raussens V, Ruysschaert JM, Goormaghtigh E (2004) Analysis of H-1/H-2 exchange kinetics using model infrared spectra. ApplSpectrosc 58:68–82
